# Osteitis Condensans Ilii: An Uncommon Cause of Back Pain

**DOI:** 10.7759/cureus.4518

**Published:** 2019-04-22

**Authors:** Sharmi Biswas, Venu Madhav Konala, Sreedhar Adapa, Pravallika Amudala, Srikanth Naramala

**Affiliations:** 1 Pediatrics, Weill Cornell Medical College, New York, USA; 2 Internal Medicine, Ashland Bellefonte Cancer Center, Ashland, USA; 3 Nephrology, The Nephrology Group, Visalia, USA; 4 General Medicine, Kakatiya University, Warangal, IND; 5 Rheumatology, Adventist Medical Center, Hanford, USA

**Keywords:** osteitis condensans ilii, sacroilitis, peripartum back pain

## Abstract

Osteitis condensans ilii (OCI) is a benign cause of low back pain, which is self-limiting. Though OCI is still an orthopedic mystery, mechanical stress across the joint is a significant triggering factor according to the prevailing theories. The traditional location of involvement is around the ileum, and can be misinterpreted as sacroiliac joint (SIJ) involvement. We present a case of bilateral OCI with sclerosis based on radiological finding in a 30-year-old female presenting with chronic low back pain.

## Introduction

Osteitis condensans ilii (OCI) is one of the benign etiologies of chronic axial low back pain. Most of the time, OCI is an incidental finding on plain X-ray with ileal sclerosis. OCI is predominantly found in women of childbearing age in the prepartum or postpartum period. It can also present in nulliparous women and men [1]. It is commonly misdiagnosed as sacroiliitis, with the etiology being around the Ileum [2]. In general, OCI is asymptomatic, but in a few patients, it manifests with low back pain at a young age which mimicks axial spondyloarthropathy (SpA) [3]. Etiologies involving sacroiliac joints (SIJs) needs to be considered in differential diagnosis [4]. A case of bilateral iliac sclerosis with OCI is discussed here with a mini literature review.

## Case presentation

**Figure 1:**
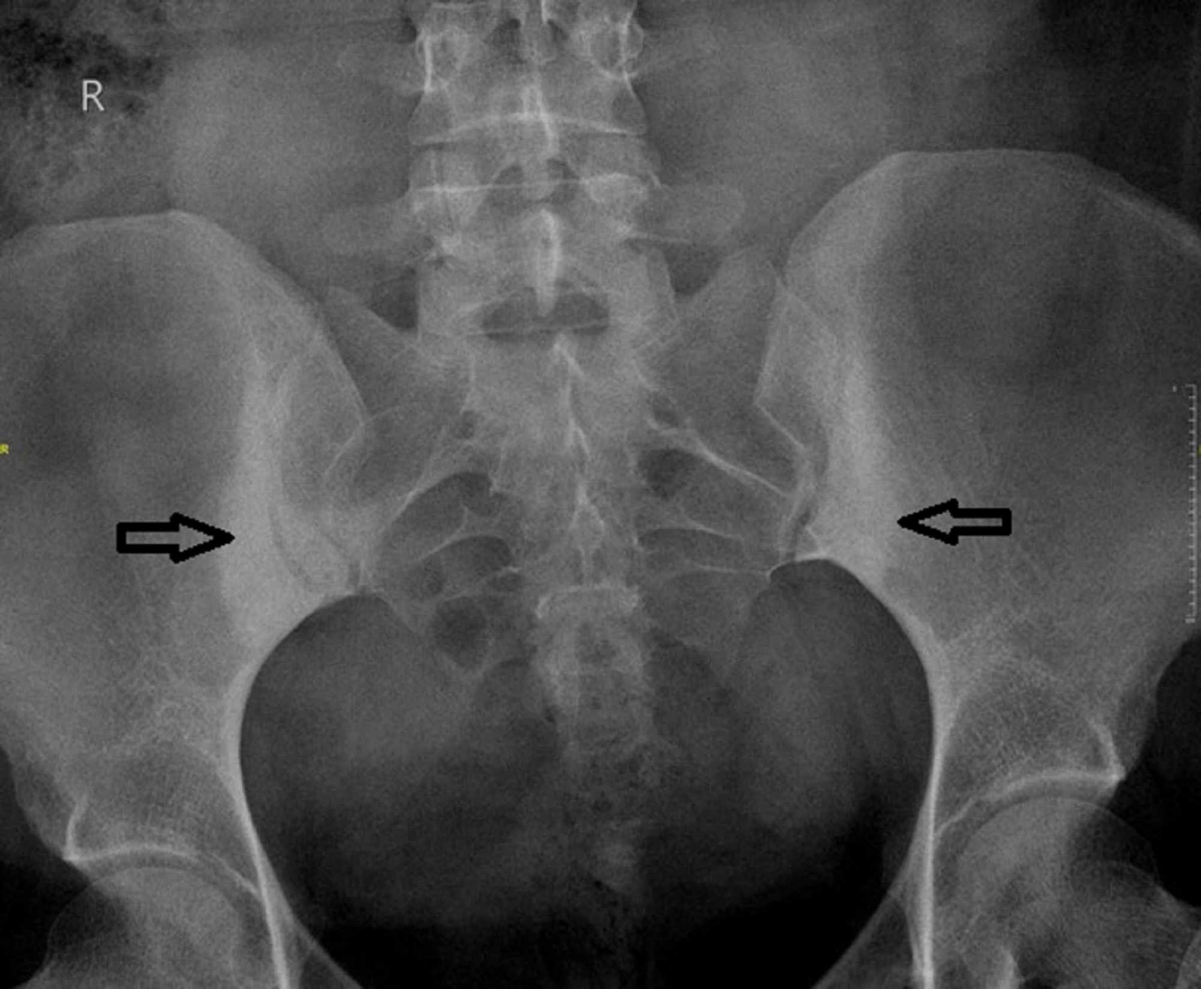
X-ray of the sacroiliac joint Anterior-posterior view showing significant sclerosis at the iliac border of the sacroiliac joints.

A 30-year-old Hispanic female referred to our clinic with the history of chronic low back pain for the last two years. She mentioned that she has been suffering from pain in her back during her second peripartum period which never resolved. During her first pregnancy, she had this pain, but it resolved after delivery. Both of her pregnancies were normal vaginal deliveries without any complications five years apart. Now her pain is mostly in the lower lumbosacral area, sharp in character, 10/10 in intensity intermittently. It gets aggravated by lifting heavy weights or doing household chores and relieved by taking rest. She was taking ibuprofen and cyclobenzaprine as needed, with symptomatic relief. She denied any joint stiffness, swelling, deformity, no skin rash, no eye, and bowel symptoms. She denied any weight loss. Physical examination was normal. Faber’s /Patrick’s test (flexion, abduction, external rotation of hip) was non-revealing. Straight leg raising test and Schober’s test were normal. X-ray of her back showed significant sclerosis at the iliac border of SIJs (Figure 1). Several other tests were performed to exclude inflammatory and other pathologies (Table 1). No other sources of inflammation were identified. She had physical therapy for six weeks and was recommended to continue exercises at home. She was also prescribed Ibuprofen and cyclobenzaprine as needed at the same time. She had significant improvement in her symptoms in three months with minimal usage of Ibuprofen and completely stopped cyclobenzaprine.

**Table 1:** Laboratory parameters HLA: human leukocyte antigen; ESR: erythrocyte sedimentation rate; PCR: polymerase chain reaction; GC: gonorrhea.

HLA B27	Negative
Calcium	9.1 mg/dl
Phosphorus	3 mg/dl
C- reactive protein	<0.6 mg/dl
ESR	8 mm/hr
Alkaline phosphatase	70 IU/L
Chlamydia trachomatis by PCR	Not detected
GC Gonorrhea by PCR	Not detected

## Discussion

OCI is a rare etiology of chronic low back pain which has an incidence rate of 0.9%-2.5%, mostly among women in the prepartum and postpartum period [1]. Initially, OCI was considered as one variety of ankylosing spondylitis though human leukocyte antigen (HLA) B27 is predominantly negative among the patients. Hallmark of OCI is sclerosis of the articular portion of the iliac bone. Despite some exceptional cases, OCI is generally not associated with elevated inflammatory markers and hence not classified as inflammatory arthritis [2].

Still, the pathophysiology of OCI is not clearly understood, but the increased mechanical stress on ileum is considered one of the causative factor of OCI in pregnant women. The increased vascularity in the ileum leads to remodeling of bone causing sclerosis. Histopathology of the sclerosed bone showed increased lamellar bone in biopsies of the affected region [4]. In general, OCI is asymptomatic, but in a few patients, it manifests with low back pain at a young age which mimicks axial SpA [3]. The pain of OCI might radiate to the bilateral gluteal area and posterior aspect of the thighs along with positive SIJ tenderness as confirmed by a positive Faber's test. The diagnosis of OCI is commonly made on radiological findings which should be differentiated from spondyloarthropathies, inflammatory arthritis, and malignancy. A typical radiological finding to distinguish OCI from other sacroiliac abnormalities is the triangular shape of sclerosis at the iliac border with preserved joint space. Management involves physical therapy and the use of non-steroidal anti-inflammatory drugs and muscle relaxants as needed. Though OCI symptoms are self-limiting and even radiological findings can disappear with time, it is essential to diagnose OCI as refractory cases can cause a varying degree of disabilities and might require surgical intervention [1,5].

## Conclusions

Physicians should be aware of this rare benign self-limiting disease and should differentiate it from other sacroiliitis mimics which helps in establishing an accurate diagnosis promptly, thereby avoiding inappropriate treatment and potential toxicities.
